# Perception survey on the introduction of clinical performance examination as part of the national nursing licensing examination in Korea

**DOI:** 10.3352/jeehp.2017.14.26

**Published:** 2017-10-25

**Authors:** Su Jin Shin, Yeong Kyeong Kim, Soon-Rim Suh, Duk Yoo Jung, Yunju Kim, Mi Kyoung Yim

**Affiliations:** 1 College of Nursing, Ewha Womans University, Seoul, Korea; 2 College of Nursing, Catholic University of Pusan, Korea; 3 College of Nursing, Kyungpook National University, Daegu, Korea; 4 Department of Nursing, JEI University, Korea; 5 Research and Development Division, Korea Health Personnel Licensing Examination Institute, Seoul, Korea; Hallym University, Korea

**Keywords:** Clinical performance examination, Focus groups, Korea, Nursing licensure, Surveys and questionnaires

## Abstract

**Purpose:**

The purpose of this study was to analyze opinions about the action plan for implementation of clinical performance exam as part of the national nursing licensing examination and presents the expected effects of the performance exam and aspects to consider regarding its implementation.

**Methods:**

This study used a mixed-methods design. Quantitative data were collected by a questionnaire survey, while qualitative data were collected by focus group interviews with experts. The survey targeted 200 nursing professors and clinical nurses with more than 5 years of work experience, and the focus group interviews were conducted with 28 of professors, clinical instructors, and nurses at hospitals.

**Results:**

First, nursing professors and clinical specialists agreed that the current written tests have limitations in evaluating examinees’ ability, and that the introduction of a clinical performance exam will yield positive results. Clinical performance exam is necessary to evaluate and improve nurses’ work ability, which means that the implementation of a performance exam is advisable if its credibility and validity can be verified. Second, most respondents chose direct performance exams using simulators or standardized patients as the most suitable format of the test.

**Conclusion:**

In conclusion, the current national nursing licensing exam is somewhat limited in its ability to identify competent nurses. Thus, the time has come for us to seriously consider the introduction of a performance exam. The prerequisites for successfully implementing clinical performance exam as part of the national nursing licensing exam are a professional training process and forming a consortium to standardize practical training.

## Introduction

The Korean government has increased the number of nursing educational institutions and their recruitment quota since 2010 as a means to alleviate the shortage of nursing personnel in small regional hospitals; this has resulted in a dramatic increase of nursing students, which puts the practicum courses of many schools in a questionable state, since many hospitals are not available for student practice [1]. Therefore, an educational curriculum focused on nurses’ actual jobs and processes that can verify a nurse’s integrated practical ability are increasingly necessary.

With the growing need for the verification of health care providers’ practical abilities, more domestic national examinations for health care providers are introducing performance exams [2]. Performance exams have already been implemented in a wide range of national examinations, including exams for medical doctors, paramedics, dental hygienists, and dental technicians, as well as preliminary exams for doctors and dentists who graduated from foreign universities. For medical doctors, a clinical performance examination using a standardized patient has been part of the national examination since 2010, and it will also become a part of the national examination for dentists starting in 2021 [3]. Regarding performance exams for domestic nurses, the Korean Accreditation Board of Nursing Education (KABONE) has a core nursing skills evaluation test [4], which aims at measuring learners’ achievements, as a part of their accreditation process. Abroad, a performance exam for nurse licensure can be found in Canada; Quebec has a performance exam consisting of 16 items, and it is carried out with simulated patients [5].

However, the national nursing licensing examination has roughly 20,000 annual applicants, which means that introducing a performance exam as part of the licensing examination is not a simple matter. Therefore, in-depth discussions and a general consensus on the necessity of a performance exam, as well as viable methods of introducing it, must precede its implementation. For this reason, this study will explore the necessity and viability of introducing a performance exam through an investigation and analysis of the opinions of nursing education experts and field experts. Its specific research purposes are as follows:

(1) To understand opinions about the necessity of introducing a performance exam and the possible action plans for its implementation.
(2) To understand the expected effects of introducing a performance exam and aspects to be considered in this process.

## Methods

### Study design

This study used a mixed-methods design combining quantitative and qualitative approaches. First, a perception survey was conducted using a questionnaire; targeting nursing professors and clinical nurses with more than 5 years of work experience, the survey investigated opinions on the desirable aspects of a performance exam and operational plans, such as ways to implement a performance exam, advisable forms of the test, abilities that need to be tested, nursing skills to be evaluated, ways to manage the test, and when the test should be administered.

The second method was to conduct focus group interviews (FGIs). Using a semi-structured questions, the opinions of nursing education experts—college professors, clinical instructors, and on-site nurse leaders at hospitals—were investigated in depth.

### Data collection methods

***Questionnaire survey:*** The questionnaire survey targeted nursing college professors, clinical nurses, and experts in nursing education policy. Among the institutions of nursing education and nursing policy (200 nursing colleges, 20 general hospitals with more than 300 wards, and KABONE), the survey selected its respondents by convenience sampling based on the regional distribution of nursing colleges and general hospitals. The survey took 20–30 minutes to complete, and participants were rewarded with gifts worth 10 dollars. Roughly 250 copies of the questionnaire were distributed, 202 were collected, and 200 were analyzed, since 2 of them were deemed not to contain sincere responses.

Our research team analyzed the subjects of foreign national examinations (including performance exams), the status of domestic practical training, and the status of the core nursing skills training/education presented and validated by KABONE [4], and the questionnaire about the content and methods of a performance exam was developed on this basis. The items in the questionnaire were as follows: validity of the nursing skills to be included in the test, the test implementation method, the most preferable type of the test, abilities to be evaluated, the operating mode of the test, the timing of test implementation, the number of evaluators, public release of test items, a reasonable test fee, the proper number of questions, and the passing criteria. The validity of the nursing skills to be included in the test was evaluated on a 4 point Likert scale: 1, not valid at all; 2, not valid; 3, valid; 4, very valid, and the Cronbach’s alpha was 0.92.

***Focus group interviews:*** FGIs were conducted in order to collect the opinions of practitioners such as nurses, professors, and clinical instructors. FGIs were done with 28 participants, all of whom were professors with equal to or more than 2 years of teaching experience or clinical nurses with equal to or more than 5 years of work experience from various regions. Every group consisted of professors, clinical instructors, and hospital nurse leaders, who were all interviewed by a researcher. Each interviewee received an explanation of the purpose of the study, the process, and the methods of data collection before the interview, and they were all notified of their right to refuse further participation at any time during the interview. They all submitted a written consent form. Data were collected from March to June 2013 in the form of recorded audio files, transcripts of the recordings, and post-interview filed notes written by researchers. The following semistructured questions were asked in the FGIs:

(1) What are your thoughts on the introduction of a performance exam as part of the nurse licensing examination?
(2) What are the expected effects of introducing a performance exam?
(3) What are the obstacles to introducing a performance exam?
(4) What content should be included on a performance exam?
(5) Which format should a performance exam have?
(6) What ramifications would introducing a performance exam have on nursing education?

### Data analysis methods

Using IBM SPSS ver. 19.0 (IBM Corp., Armonk, NY, USA), the survey results were analyzed in terms of frequency, percentage, mean value, and standard deviation. The qualitative data collected through FGIs were categorized and the themes were extracted through content analysis.

### Ethical approval

In terms of ethical considerations regarding the research participants, the survey and the interview were conducted only when the participant understood the purpose of the research and voluntarily signed a written informed consent form to participate in the research. The written consent form guaranteed the participant’s anonymity, and explained that all data would be used only for research and would be destroyed after the study.

## Results

### Survey results

***General characteristics of the participants:*** Of 200 survey participants, 50% were college professors, and 39.1% were clinical nurses. The participants’ average age was 44.34 years, average teaching career 12.42 years, and average clinical career 10.61 years. In terms of geographical distribution, 19.3% of them worked in Seoul, 11.9% in Gyeonggi Province, 14.4% in Chungcheong Province, and 31.2% in Gyeongsang Province (Table 1). Raw data were available from Supplement 1.

***Validity of the nursing skills to be included in the clinical performance examination:*** The nursing skills to be included in the performance exam were evaluated on a 4-point Likert scale, and the item that scored the highest validity was measuring vital signs (mean, 3.53; standard deviation, 0.77), followed by indwelling catheter insertion, endotracheal suction, intramuscular injections, nasal cannula oxygen therapy, catheterization, subcutaneous injections, enteral nutrition via nasoenteric tubes, oxygen saturation measurements and electrocardiogram monitoring, basic cardiopulmonary resuscitation and defibrillator use, and intravenous therapy (Table 2).

***Action plans for the clinical performance exam:*** Of the respondents, 71.3% chose a hands-on performance exam on simulators or standardized patients as the most suitable format for the performance exam, and for proper type of the test, common choices were an objective structured clinical examination (27.8%), a scenario-based nursing performance examination (37.4%), and a clinical performance examination using standardized patients (32.3%). When multiple answers were allowed, the important abilities that many thought needed to be evaluated were basic nursing skills (83.2%), critical thinking skills (56.4%), communication skills (55.9%), and clinical reasoning skills (54.5%).

Under the assumption that a performance exam will be developed and implemented, many chose across-the-board administration of the test managed by a central institution (the Korea Health Personnel Licensing Examination Institute) as the most preferable form of test management (54.9%). Regarding the proper timing for the test, ‘after the written test’ received the most votes (49.5%), but ‘all year round’ also received many votes (32.3%) (Table 3).

### Results of the focus group interviews

***General characteristics of the participants:*** The participants in the FGIs consisted of 28 professors, including clinical instructors and nurses at hospitals. Their ages ranged from 32 to 55, clinical experience from 2 to 30 years, and teaching career from 3 to 10 years; overall, the participants showed considerable variation in their experiences.

***Main results:*** Both supporting and opposing views on the implementation of a performance exam were articulated, and the main results are presented in Table 4.

(1) Views supporting the implementation of clinical performance exam

Regarding the expected effects of introducing clinical performance exam, there were high hopes about the enhanced clinical and practical abilities of new nurses who will pass the national examination. The new recruits will have to undergo extensive training while preparing for the performance exam, which will improve their clinical performance more than preparing for the current examination. These improvements in their clinical abilities will then boost their adaptability to clinical situations, which will help reduce the turnover intention of new nurses who have worked for less than a year.

However, schools with practicum courses anticipated that the performance exam would bring about the standardization of nursing practice training, which means that the improvement of the evaluation system through more serious high-stakes testing will lead to the standardization of practice courses. This means that the performance exam will change our current knowledge-oriented curriculum into a more harmonious one that balances knowledge, attitudes, and techniques. Additionally, infrastructure for the performance exam will be established in each educational institution as part of preparing for the exam; thus, the quality of nursing schools in general will be improved. Furthermore, interconnections between educational institutions and clinical practitioners will be strengthened, as ensuring a higher test passing rate will require institutional instructors and leaders of clinical institutions to develop a keener interest in teaching the students. The participants’ statements about the aforementioned points are presented below.

< Improvement of new nurses’ clinical performance abilities>

“If a performance exam is introduced, it seems that new nurses’ proficiency in nursing skills will increase faster than before. I think that your other work-related abilities can be improved only when you have the necessary nursing skills.” (Participant 3, professor, age 43)

< Increasing new nurses’ adaptability to clinical situations>

“The ultimate thing that keeps the students from quitting their job at a hospital, when they’re out of school, is their core nursing skills. I think that maybe we need to evaluate them somehow.” (Participant 13, professor, age 37)

< Standardization of nursing practice training>

“Standardization! Every hospital and every ward has a different protocol, and it’s so prevalent (every hospital has different nursing procedures). I think that getting them (different nursing techniques) standardized might be the most important effect of introducing the performance exam.” (Participant 10, professor, age 45)

<Improving the balance of nursing education: knowledge, attitudes, techniques>

“If a performance exam becomes part of the national exam, then it will be reflected in every subject that needs testing…It would be a game-changer, wouldn’t it? I think it will ultimately reinforce the education on techniques and attitudes.” (Participant 28, chief nurse, age 41)

<Establishing the necessary infrastructure at educational institutions>

“I expect that the infrastructure will be established. If a performance exam is included in the national exam, then schools probably will start investing for that purpose. The system creates the basis for expanding the infrastructure, so to speak.” (Participant 23, chief nurse, age 42)

<Strengthening interconnections between educational institutions and practice institutions>

“If a performance exam becomes part of the national exam and if it’s institutionally necessary, then I think that clinical leaders will change their attitudes towards practice instruction. Wouldn’t they feel (more) responsible?” (Participant 14, chief nurse, age 54)

(2) Views opposing the implementation of a performance exam Some universities already have a graduation qualification system, the purpose of which is to manage a balanced nursing curriculum in which nursing students’ clinical performance, skills, and attitudes are improved. Some respondents also pointed out that the accreditation test for nursing education held by KABONE already evaluates nursing students’ core nursing skills, and therefore could be an alternative to an official performance exam; as such, the test might be pointless. In addition, many were concerned that integrating the test into the national exam would significantly increase the burden on institutional instructors as well as on students; in particular, some faculty members in charge of specific courses are more involved in practical training than other faculty members, meaning that the burden will be concentrated on a few professors. Practical examinations tend to cause more stress than knowledge-oriented examinations, and students may fail the national exam due to a single mistake; the graduating seniors would inevitably feel high levels of stress in such circumstances. The participants’ statements about the aforementioned points are presented below.

< The accreditation test for nursing education is sufficient>

“If we’re talking about 20 really simple nursing skills, universities can make students take the accreditation test. Otherwisse, we can do it with a graduation qualification system.” (Participant 24, professor, age 32)

< Increased burden on educators>

“I think many colleges might be preoccupied with nothing but the national exam because a performance exam can be really burdensome. In fact, the reality of our clinical training is that not many instructors are in charge of the training, and there are few opportunities where students can see and learn from actual patients. Finding a solution to that can be really taxing for universities.” (Participant 18, nurse, age 40)

< Meaninglessness of the performance exam>

“Even the performance exam for advanced practice nurses had a low level of difficulty, and there were so many problems that they had to change the performance exam to essentially be a written test. I doubt that an undergrad-level nursing performance exam is necessary.” (Participant 12, nurse, age 36)

(3) Aspects to consider if a performance exam is introduced The first point to be considered when introducing a performance exam as part of the national exam is that efforts first need to be made to ameliorate the quality of clinical training. When students’ main activity in a clinical training institution is observation instead of handson practice, the students cannot be effectively ready for the performance exam. Therefore, cooperation from clinical training institutions must precede the implementation; they are in charge of most clinical training processes, and they have the responsibility of cultivating the students’ critical thinking skills and good judgment.

Moreover, the national nursing licensing examination is different from other health-related exams in that it has significantly more examinees; the examinees need to be effectively managed so that they will not be at any disadvantage. In addition, the assessment content of the performance exam needs to be standardized, and the evaluators also need to be trained so that their assessments will be considered credible and valid, which is paramount.

< Efforts to ameliorate clinical training>

“The nurse educator’s role is important. We get loads of work from the hospital already, and student practical training on top of that…I wish there was some systematic reward. I think the reward has to be officially institutionalized.” (Participant 8, professor, age 42)

<Fulfilling the necessary conditions for the effective management of examinees>

“There are too many people. Concrete measures must be taken for us to be able to accommodate these people. Facilities, equipment for implementing the performance exam, and the cost: these will be huge problems.” (Participant 22, professor, age 39)

<Standardization of the assessment content of the performance exam>

“Every college has a different environment for student practice. And if every hospital has a different standard for nursing skills, there will be chaos when a performance exam is implemented. What has to come first is the standardization of nursing skills, based on which the assessment content of the performance exam must be standardized.” (Participant 24, professor, age 32)

<Ensuring the credibility and validity of the evaluators of the performance exam>

“Having many examinees requires many evaluators. Every evaluator needs to apply the same criteria, so I think that ensuring the evaluators’ credibility and validity will be the most important task.” (Participant 2, nurse, age 36)

## Discussion

This study analyzed nursing experts’ opinions on the introduction of a performance exam, which will be an important reference point when an action plan is made for implementation. This study also examined the anticipated effects of a performance exam and opinions about alternatives in case the test is not implemented, which will serve as a reference point for analyzing the advantages and disadvantages of introducing a performance exam as part of the national nursing examination. Based on our results, we will now discuss the necessity of a performance exam, its benefits, and an action plan for development and implementation, as well as its limitations.

### Necessity and benefits of introducing a performance exam

We found that nursing professors and clinical experts were concerned about the limitations of the current written test as a method of evaluating examinees’ abilities, and that they had high expectations for the beneficial effects of introducing a performance exam. In particular, their hopes were highest for the effects of a hands-on performance exam on simulators or standardized patients, which was expected to improve nurses’ basic nursing skills. Still, the number of annual examinees (20,000) would be a serious challenge, as the center-controlled *en bloc* style was deemed the most preferable form of exam administration. Nevertheless, the respondents believed that a performance exam needs to be implemented after confirmation of its credibility and validity, provided the test is appropriate for evaluating nurses’ abilities and facilitating skill improvement. Therefore, the groundwork for the smooth implementation of the test seems to be the next necessary step; this would include preparatory work such as securing physical resources and building a consortium of educators and hands-on workers. Moreover, the introduction of a performance exam will lead to the construction of infrastructure geared to the test in educational institutions, and will create an opportunity for nursing schools to improve the overall quality of their practical training, as indicated by the effect analysis report of clinical skills assessments for medical doctors [6]. According to that report, the introduction of a performance exam in medical doctors caused positive changes such as increased participation by students in the practical training encouraged by professors; additionally, resources were efficiently utilized and regional consortiums were vitalized, all leading to the improvement of clinical techniques, attitudes to patients, confidence, and communication skills among interns and residents [7]. Another report reported that mandating 4 years of clinical performance examinations for nursing students, in comparison to written tests, increased the chance of evaluating the students’ clinical and communication skills [8]. Considering these reports, it seems evident that continuous assessment and management are the keys for enhancing clinical and communication skills.

Nonetheless, participants articulated other opinions as well, arguing that the quality of education would be guaranteed only if the education stays faithful to the curriculum and if the KABONE accreditation process functions properly. According to a perception survey on the accreditation test for nursing education, professors in charge of basic nursing education thought that the accreditation test was necessary for the physical environment of practical training to be ameliorated [9]. This means that the institutional apparatus plays an important role in creating the basis for practical training. Therefore, it is necessary to analyze how much the practical abilities of nursing students changed after the implementation of the accreditation evaluation (by a meta-evaluation of the accreditation evaluation), and if it turns out that the accreditation system does not guarantee a sustainable education, remedies will have to be made to complement the written test.

### Action plans for a performance exam as part of the national nursing licensing examination

Most respondents chose a hands-on performance exam using simulators or standardized patients as the most suitable format for a performance exam as part of the national nursing licensing exam. This seems to reflect the positive outcomes of core nursing skill education reinforced by KABONE, and the perspective that firsthand training is more fitting to the nature of a nursing job than virtual-reality based training. The skill assessment test for medical doctors—which is made up of 12 exam subjects—has 3,500 annual candidates. The performance exam for nurses, in contrast, has roughly 20,000 annual examinees; there must be regional centers that can accommodate these people and increase the total capacity before implementation. Additionally, this study indicates that the performance exam should be held multiple times a year, and if we accept the opinion that examinees should have multiple chances to take the test, it is advisable to maintain the test system on an all-year-round basis.

The exam subjects need to comprise items that evaluate not simple skills, but problem-solving skills in complicated situations; only then will the test be able to accomplish its high-level goals. Additionally, considering the target abilities of the performance exam, the test must be a clinical performance examination requiring a complex approach to standardized patients or simulators. If a part of the purpose of the test is to increase the opportunity to train for the practical skills required on the job, it is desirable to allow a retake when a candidate fails the test.

In preparation for the introduction of a clinical performance exam as part of the national nursing licensing examination, this study examined the necessity of a performance exam and possible action plans for implementation. In conclusion, the current national examination is perceived to be limited in its ability to select nurses who are well-qualified for the changing medical environment, and thus, introducing a clinical performance exam as part of the national nursing licensing examination is expected to improve nurses’ work ability. Many opinions collected in this study simultaneously present hopes, anticipated problems, and possibilities regarding the prerequisites for a performance exam.

The nursing education process must take 2 preparatory steps: improving the quality of clinical practice training, and standardizing the assessment content of a performance exam. To these ends, we suggest establishing a consortium for standardizing the practical training of over 200 nursing schools. Medical schools will be a good reference; they established regional consortiums, through which they cooperatively developed the assessment content of the performance exam before it was implemented. When the test is implemented, a sufficient number of evaluators will have to be secured and trained, and plans for the effective management of examinees will be necessary. The Korea Health Personnel Licensing Examination Institute needs a taskforce to prepare for the year-round administration of the test.

## Figures and Tables

**Table 1.** General characteristics (N=200)

Characteristic	Value
Age (yr)	44.34 ± 7.45
Clinical experience (yr)	12.42 ± 8.75
Educational experience (yr)	10.61 ± 8.86
Region	
Seoul	39 (19.3)
Gyeonggi	24 (11.9)
Gangwon	19 (9.4)
Chungchung	29 (14.4)
Gyeongsng	63 (31.2)
Jeola or Jeju	25 (12.4)
Affiliation	
University	101 (50.0)
Hospital	79 (39.1)
Institution related to education or policy	18 (8.9)
Other	2 (1.0)

Values are presented as mean±standard deviation or number (%).

**Table 2.** Validity of the nursing skills evaluated on clinical performance exam

No.	Nursing skills	Mean ± standard deviation
1	Measuring vital signs	3.53 ± 0.77
2	Indwelling catheter insertion	3.51 ± 0.72
3	Endotracheal suction	3.49 ± 0.80
4	Intramuscular injections	3.49 ± 0.78
5	Nasal cannula oxygen therapy	3.42 ± 0.80
6	Catheterization	3.41 ± 0.74
7	Intradermal injections	3.41 ± 0.79
8	Subcutaneous injections	3.40 ± 0.80
9	Enteral nutrition via nasoenteric tubes	3.38 ± 0.74
10	Oxygen saturation measurement and electrocardiogram monitoring	3.37 ± 0.79
11	Basic cardiopulmonary resuscitation and defibrillator use	3.36 ± 0.91
12	Intravenous therapy	3.35 ± 0.92
13	Oral medication	3.26 ± 0.89
14	Transfusion therapy	3.21 ± 0.99
15	Postoperative nursing care	3.21 ± 0.82
16	Preoperative nursing care	3.13 ± 0.86
17	Use of tracheotomy tube	3.05 ± 0.95
18	Enema	3.04 ± 1.04
19	Isolation precautions: personal protective equipment and waste management	2.97 ± 1.00
20	Admission and discharge care	2.82 ± 0.99

**Table 3.** Perceptions of the action plans for implementing clinical performance exam

Variable	Category	No. (%)
Mode of implementation	Written performance exam	19 (9.4)
	Performance exam using virtual reality simulations	39 (19.3)
	Hands-on performance exam using dummies or standardized patients	144 (71.3)
Abilities to be evaluated	Critical thinking skills	114 (56.4)
	Communication skills	113 (55.9)
	Clinical reasoning skills	110 (54.5)
	Basic nursing skills	168 (83.2)
Mode of management	Administered at individual universities first, then certified	41 (21.0)
	Administered en bloc through a central institution (KHPLEI)	107 (54.9)
	Some portions administered at universities, other portions at a central institution (KHPLEI)	39 (20.0)
	Other	8 (4.1)
Time of implementation	Before the written test	32 (16.2)
	After the written test	98 (49.5)
	Whenever appropriate, regardless of the written test	64 (32.3)
	Other	4 (2.0)
No. of evaluators	1 Evaluator per applicant	12 (6.1)
	2 Evaluators per applicant	140 (70.7)
	3 or more evaluators per applicant	39 (19.7)
	Other	7 (2.5)
Release of test items	Release all items	20 (10.0)
	Release test items only	21 (10.5)
	Release test items and offer relevant guidelines	153 (76.5)
	Release not needed	6 (3.0)

KHPLEI, Korea Health Personnel Licensing Examination Institute.

**Table 4.** Qualitative results from focus group interviews

Supporting views	Opposing views	Pre-implementation considerations
Improvement of new nurses’ clinical performance	Accreditation test for nursing education suffices	Efforts to ameliorate clinical training
New nurses’ increased adaptability to clinical situations	Increased burden on educators	Effective management plan necessary for exam applicants
Standardization of nursing practice training	Meaninglessness of performance exam	Standardization of performance exam’s assessment content
Quality improvement of nursing education: more balance		Development of questions that evaluate multiple abilities
Expansion of basic resources for educational institutions		Ensuring the credibility and validity of the evaluators (sufficient training of the evaluators)
Strengthened interconnections between educational institutions and practice institution		

## Supplementary materials

Supplement 1. Data file is available from https://doi.org/10.7910/DVN/E83CGP.

Supplement 2. Audio recording of the abstract.
